# AIIMS ICU Rehabilitation (AIR): development and description of intervention for home rehabilitation of chronically ill tracheostomized patients using the TIDieR checklist

**DOI:** 10.12688/wellcomeopenres.19340.1

**Published:** 2023-06-30

**Authors:** Swagata Tripathy, Asha P. Shetty, Upendra Hansda, Nanda Kumar P, Alok Kumar Sahoo, Mahalingam V, Sujata Mahapatra, Jayanta Kumar Mitra, P Bhaskar Rao, Kasturi Sanyal, Itimayee Panda, Guruprasad N, Jagannath Sahoo, Helen Eborral, Nazir Lone, Rashan Haniffa, Abi Beane

**Affiliations:** 1 Anesthesia & Critical Care, AIIMS Bhubaneswar, Bhubaneswar, Odisha, 751019, India; 2 College of Nursing, AIIMS Bhubaneswar, Bhubaneswar, Odisha, 751019, India; 3 Trauma and Emergency, AIIMS Bhubaneswar, Bhubaneswar, Odisha, 751019, India; 4 Neurosurgery, AIIMS Bhubaneswar, Bhubaneswar, Odisha, 751019, India; 5 Physical medicine and Rehabilitation, AIIMS Bhubaneswar, Bhubaneswar, Odisha, 751019, India; 6 Critical Public Health, The University of Edinburgh, Edinburgh, Scotland, UK; 7 Critical Care, Oxford University C S Lewis Society, Oxford, England, UK; 8 Critical Care, MORU Thailand, THailand, Thailand; 9 NICST, Colombo, Sri Lanka

**Keywords:** Intervention, Programme, Experience Based Codesign, Participatory design, TIDieR, Homecare, Carer, Caregiver, Tracheostomy, Chronically ill, Chronically Critically Ill.

## Abstract

**Background**: The paucity of state-supported rehabilitation for chronically ill patients with long-term tracheostomies has ramifications of prolonged hospital-stay, increased burden on acute-care resources, and nosocomial infections. Few interventions describe home rehabilitation of adult tracheostomized patients. Almost none involve stakeholders. This paper describes the All-India Institute of Medical Sciences (AIIMS) ICU rehabilitation (AIR) healthcare intervention developed to facilitate home rehabilitation of chronically ill tracheostomized patients.

**Methods**: The AIR intervention was developed in six stages using the experience-based codesign theory (EBCD). A core research-committee studied prevalent knowledge and gaps in the area. Patients-carer and health-care stakeholders’ experiences of barriers and facilitators to home care resulted in an intervention with interlinked components: family-carer training, equipment bank, m-health application, and follow-up, guided by the Medical Research Council (MRC) framework. Healthcare stakeholders (doctors, nurses, medical equipment vendors) and patient-carer dyads were engaged to gather experiences at various stages to form smaller codesign teams for each component. Multiple codesign meetings iteratively allowed refinement of the intervention over one year. The Template for Intervention Description and Replication (TIDieR) checklist was used to report the AIR intervention.

**Results**: The first component comprised a minimum of three bedside hands-on training sessions for carers relating to tracheostomy suction, catheter care, monitoring oxygenation, enteral feeding, skincare, and physiotherapy, buttressed by pictorial-books and videos embedded in a mobile-application. The second was an equipment-bank involving a rental-retrieval model. The third component was a novel m-health tool for two-way communication with the core group and community of other patient-carers in the project for follow-up and troubleshooting. Home visits on days 7 and 21 post-discharge assessed patient hygiene, nutrition, physiotherapy, and established contact with the nearest primary healthcare facility for the future.

**Conclusions**: Findings support the EBCD-based development using active feedback from stakeholders. Assessment of feasibility, process and effectiveness evaluation will follow.

## Introduction

With improved life support systems, acute illness is increasingly associated with patients surviving long stays in intensive care units (ICUs), prolonged hospitalization in post-acute (weaning) centres, and poor prognosis in the long term ^1^. These patients who have survived a period of acute illness, but are not yet free from life sustaining therapies are referred to as the chronic critically ill (CCI). The incidence of chronic critical illness has doubled in recent decades and may double again in the next decade ^2^.

Consensus definition for CCI combines two factors: care received in the ICU for at least eight days, and at least one out of five eligible conditions (prolonged mechanical ventilation (MV); tracheostomy; sepsis; severe wounds and/or multiple organ failure; ischemic stroke, intracerebral haemorrhage or traumatic brain injury) ^2^. Prolonged need for mechanical ventilation due to cardiopulmonary disease, inability to protect the airway due to neurological dysfunction or brain injury are the main reasons for tracheostomy in the CCI.

Since these patients are often bedridden, tracheostomized and oxygen or ventilator dependent, they require trained caregivers (CGs)for daily care such as enteral feeding, catheter care and physiotherapy. There has emerged an unmet need for adequate rehabilitative opportunities for CCI patients worldwide. In developed healthcare systems, such patients are cared for by state-delivered healthcare services—professional CGsor in nursing homes. This has a dual benefit: for the hospital, it means clearing up resources for the acutely ill, and for the patient, it means returning to familiar surroundings and a reduced risk of hospital-acquired infections. These benefits of out of hospital rehabilitation are often not available in Low Middle-Income Counties (LMICs) where state-delivered rehabilitation systems are less well established. The entire burden (physical, psychological and economical) of care often falls on family members of the patient.

The implications and outcomes (including cost) following protracted ICU stay are potentially devastating for patients and their families. Reasons for this include limited availability of and access to rehabilitation services, lower baseline health literacy, concomitant infections in the presence of antibiotic resistance, and spiralling financial burden associated with healthcare costs and loss of income.

For patients at risk of extended ICU stay and CCI, discharge home to be with family is a priority. Not only does timely discharge alleviate the burden of the cost of hospital care, but it may also reduce the incidence of healthcare associated infections (HCAIs) and provide families opportunities to be at home with their loved ones as they attempt to recover.

Although some teams from India ^3, 4^, South Africa ^5, 6^, and Thailand ^7^ have recognized that family members of chronically critically ill tracheostomized patients can be trained to accept and successfully execute home rehabilitation, the attempts have been sporadic ^8^, tailored for paediatric population ^9^, and even unsuccessful at times ^6^. Causes put forth are a lack of specific training, education and support for family CGs (hereon referred to as carers) in the form of assistive devices and timely referral to health services ^6^.

To meet this gap in Odisha, a state in Eastern India, a team of critical care physicians, nurses, rehabilitation specialists, and dieticians from the All-India Institute of Medical Sciences (AIIMS) designed an intervention to facilitate home rehabilitation of tracheostomized CCI patients. The endeavour was supported by the National Health Mission Odisha and Collaboration for Research, Training, and Implementation in Critical Care in Asia and Africa (CCAA)—a Wellcome Trust Innovations project.

The AIIMS ICU rehabilitation (AIR) project has designed an intervention that will empower and equip family carers to accept and execute home rehabilitation of chronically ill tracheostomized patients. In many instances, interventions are poorly described regarding the training material, the number of sessions, duration, dose, intensity and mode of training delivered ^10^. A detailed description of the key components of an intervention makes it easier for other researchers to replicate the intervention in clinical settings and research ^11, 12^. Thus, this paper aims to describe the novel intervention in the AIR project using the 12-item Template for Intervention Description and Replication (TIDieR) checklist ^12^.

## Methods

Mixed methods included systematic reviews ^13, 14^, qualitative studies ^7^, and data from adult tracheostomized patients in India ^8, 15^. Research on carer burden ^16^, and a cross-sectional survey (unpublished) were performed as a part of this intervention to further inform the initial development of AIR. The description of the development of the intervention using the EBCD follows. carers

### Development of the Healthcare Intervention

The AIR intervention is a bundle of components: i) Hands-on training of carers, ii) an m-Health app providing information and two-way communication via voice, message, and audio between patient-carers and the health care team, iii) an equipment bank, and iv) post-discharge home visits and telephone follow-up.

**Figure 1.**
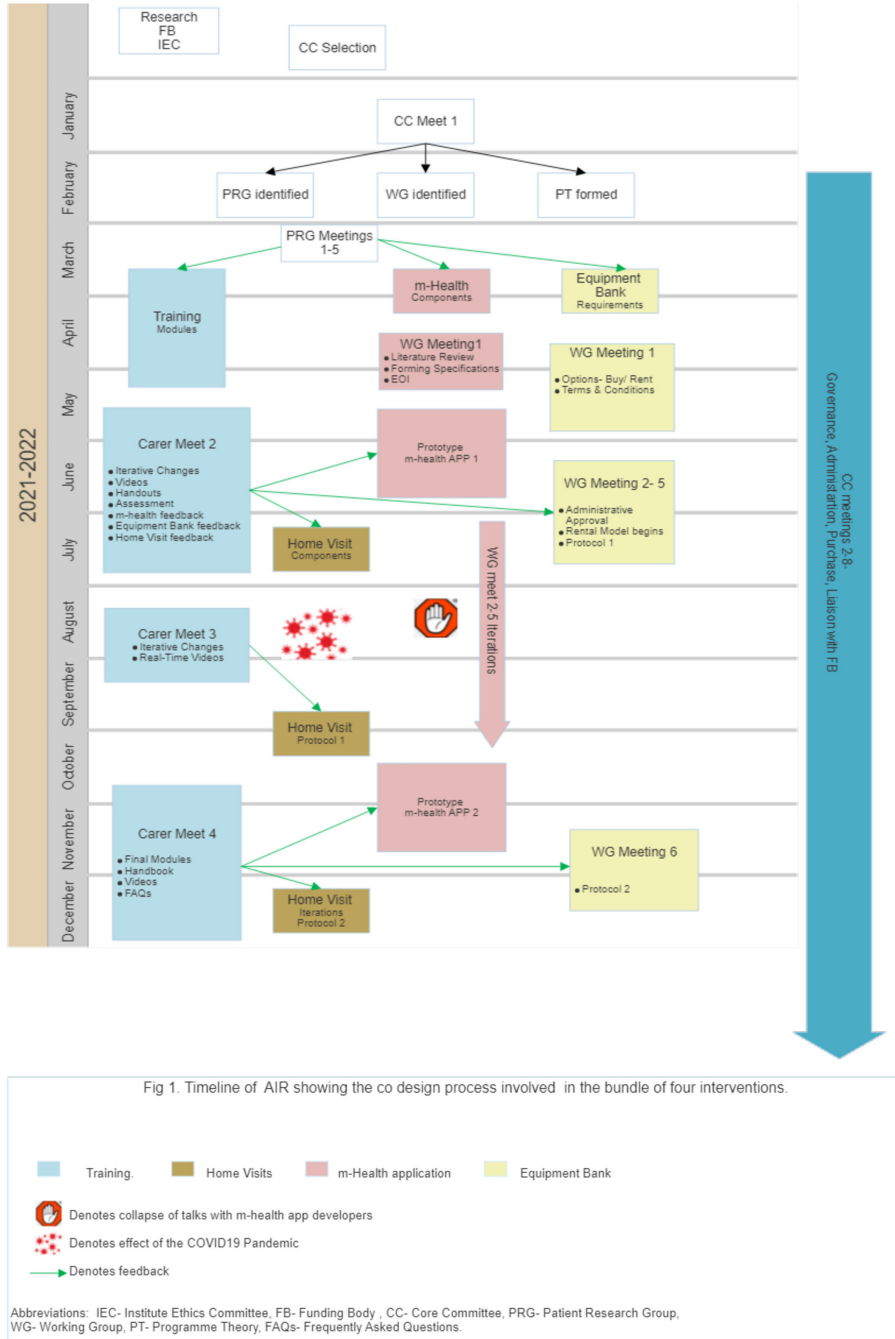
Intervention timeline.

The initial development and piloting of the AIR project followed the stages of the experience-based codesign (EBCD) method ^17– 19^. This method enables service users and providers to work collaboratively to improve care in an iterative, reflexive manner driven by stakeholders’ experiences. EBCD includes six stages: identifying the problem, collecting stakeholder (staff and patient) experiences, a series of stakeholder codesign meetings to develop the intervention, iteration of the intervention based on stakeholder feedback following implementation, and celebrating achievements by sharing the work with stakeholders. The timeline and steps of development of the AIR intervention are summarised in Figure 1.

**Figure 2.**
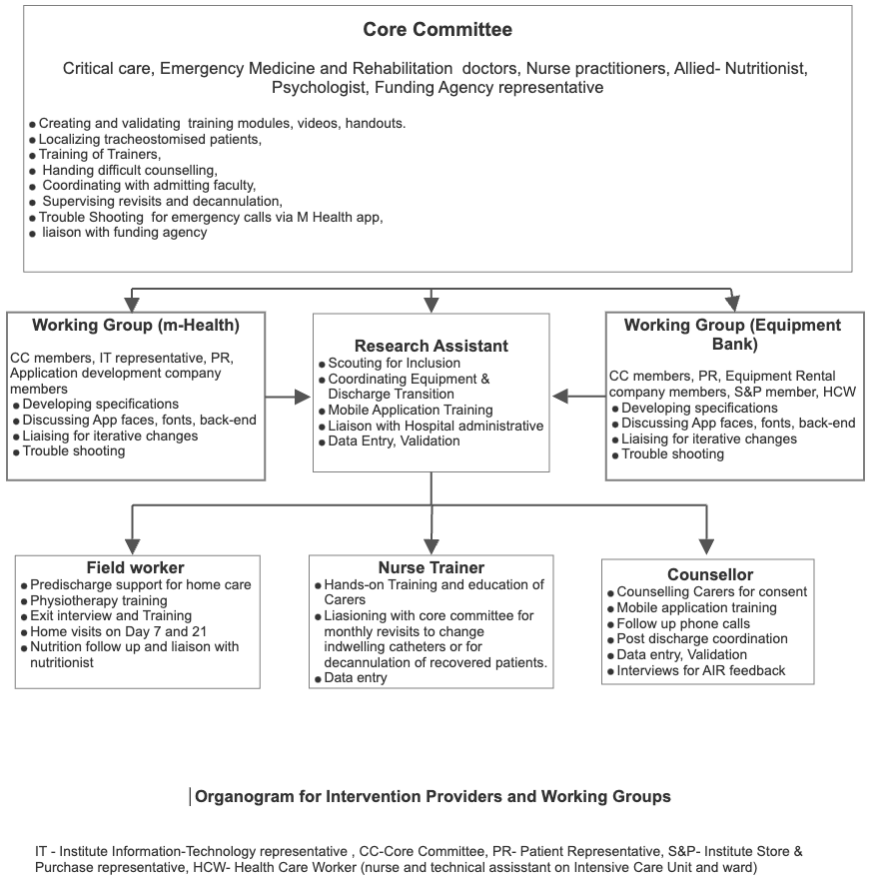
Intervention organogram.

*Stage one (identifying the problem).* We began by forming a research team (RT) consisting of doctors, nurses, and project staff (research assistant RA, Nurse Trainer NT, Psychologist P, and Field worker FW). The RT reviewed the existing literature regarding home care for tracheostomy patients ^20– 26^ and their carers ^8, 27^. Data from resource-limited settings were limited, and concurrent research was undertaken to understand the caregiver burden for home-discharged tracheostomy patients in the local set-up ^16^. We next formed a core committee (CC) that included, along with the RT, representatives of key technical members of the institute (information and technology I&T and Finance), the funding agency, and an institute staff with experience of repeated ICU admissions as the patient representative (PR). The CC was responsible for reflecting on evidence, identifying wider stakeholders, and overseeing the project. The organogram in Figure 2 and TIDieR checklist item 5 elaborates on the roles of the CC.

*Stages two and three (stakeholder engagement)* were conducted in parallel. The RT formed a Patient Representative Group (PRG) of five previously discharged tracheostomised patient-carer dyads.

Next, they mapped health care workers (HCWs) from ICU and allied specialties and non-HCW individuals likely to be involved in the patient care pathway (such as equipment providers and m-health app developers) into Working Groups (WGs). Selected members of the CC led each WG. This provided an opportunity for members of the wider multidisciplinary teams involved in the care of critically ill patients and representatives from non-medical disciplines to be involved in the design and implementation of the components of the AIR project. The WGs’ inputs resulted from engaging with the CC and sharing experiences through interviews and focus group discussions.

Finally, patient-carer dyads (participants) were screened and invited to pilot the AIR intervention. The psychologist supported them through a series of engagement exercises, including one-to-one meetings, telephone follow-up, and meeting with RT members at discharge (exit meeting).

Weekly meetings of leads of different WGs, principal investigator, and members of the CC not a part of the RT were held to allow reflexivity in the process. In addition to the engagement exercises, we disseminated information about the intervention - posters were put up in relevant areas of the hospital, and academic presentations were made to doctors, nurses, and trainees. During consent taking, a four-page handout in Odia or English was given to carers. Copies were displayed at each nursing station. A video of 10 minutes duration (in Odia, English, and Hindi) prepared by the RT was available to inform the patient-carer and clinical staff about the project to engage illiterate, visually challenged, or those who preferred to ‘see’ than read the detailed written information. The video was shareable with extended family via WhatsApp when carers needed to discuss participation in the project. Videos of members of the PRG taken during engagement exercises were shown, and eligible participants were encouraged to contact previous recruits for clarifications. Further details of materials used for stakeholder engagement are described below in TIDieR section 3, and the results of this stage of the EBCD are detailed in TIDieR Checklist item 2.

*Stage four (developing the intervention).* The research team transcribed and analyzed field notes and transcripts captured during the PRG and WG meetings. Analysis was undertaken by RT members (ST, APS, NKP, SM, UP, and AKS) using an interpretive analysis approach. The findings from different sources were triangulated to identify overarching themes and discussed with the whole CC. The critical needs identified were translated into the following interlinked interventions: training modules, m-health application, equipment bank, and post-discharge follow-up.

Work on implementing the components- the screening and training process, equipment bank, and m-health app development began by December 2020. The Home Visits began after August 2021 due to COVID-19 disruptions ( Figure 1). The design, content, and utilization of these interventions are described further in TIDieR Checklist items 3 and 4 below.

**Figure 3.**
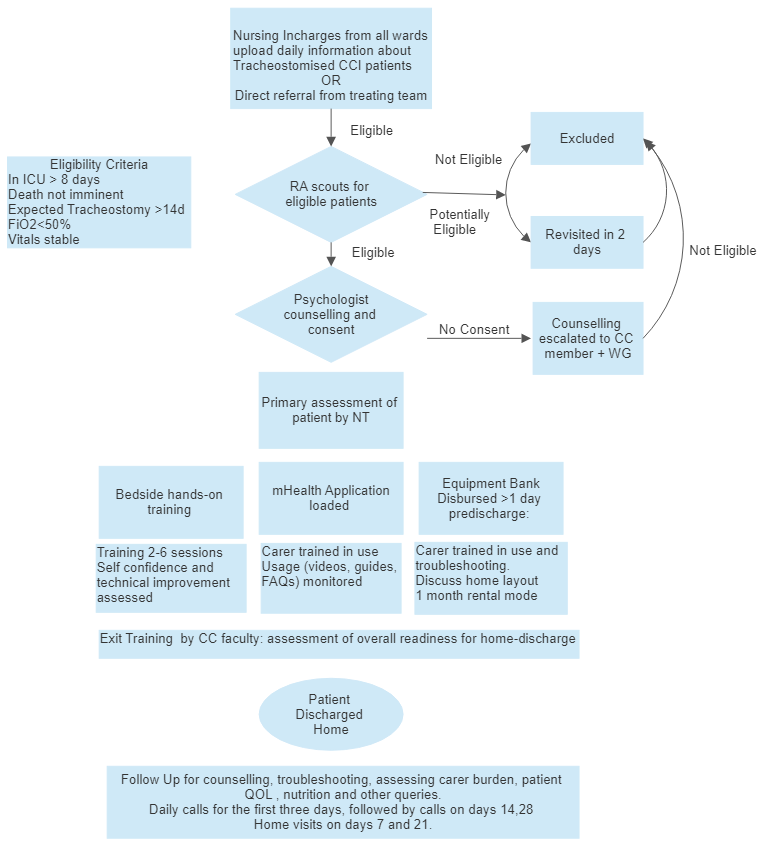
Intervention processes and procedures.

*Stage five (iteration of intervention)* was informed by multiple codesign meetings with the patient-carers, the WGs, and the CC to make iterative changes in the intervention design and take feedback on the changes implemented. Project staff telephonically and during home visits collected lived experiences from carers after home discharge. The CC formed WhatsApp groups among themselves, WGs, carers, and PRGs to engage multidisciplinary teams and allow collaborative, self-conscious criticism and appraisal of the context and subjectivity of the RT members. WhatsApp groups and the m-Health app were actively used for exchanging information, photographs, audio and video messages, suggestions, and feedback among project members- CC, WG, and Carers. One such iteration led to RT members collecting direct feedback from carers in ‘exit meetings’ at hospital discharge. ( Figure 3). The details of iterations are detailed in TIDieR Section 8,10.

*Stage six (celebrating achievements)* was undertaken with different stakeholder groups; the approach depended upon the stakeholder and stage of the project. For carers, members of the CC convened for every patient-carer dyad who returned for a change of indwelling catheters or decannulation to applaud their efforts. For HCWs, the photographs and the appreciative messages that the patients and carers sent from home on WhatsApp and mHealth chats were shared with the ICU team and referring doctors. Patient-carer dyads were invited to the ICU to meet with HCW teams on follow-up hospital visits. WG meetings moved to Zoom platform in response to the infection control practices implemented as part of the COVID-19 management. A ‘celebration event’ was organized in August 2022 on the 200 ^th^ pilot recruitment, where 40 patient-carer dyads and 60 HCWs and members of the Institute and Funding agency participated.

*Intended Sample Size-* Patients and families will be recruited into the intervention till the intervention with iterations and adaptations lends itself to an evaluation of feasibility, acceptability, and appropriateness within the project duration of two years.

### Ethics

The institutional ethical review board of AIIMS Bhubaneswar (Registration number ECR/534/Inst/OD/2014/RR20) granted ethical approval for studies informing the AIR intervention development process in AIIMS Bhubaneswar (Ref T/EMF/Anesth/ 20/53) dated 24/10/2020. All carers and patients were explained the project aims and written consent taken for elective participation, use of information sheets and photographs for academic non-commercial use.

## Results

Given the aim of this manuscript is to describe the codesign and components of the intervention, we describe the intervention components using relevant items of the TIDieR checklist. Items 3 and 4 of the TIDieR (materials and procedures) have been structured to first describe the procedures for the interconnected interventions. Then we have described the materials generated from the codesign and used (item 3) to better enable the reader to understand the methods selected in the context of the interventions delivered. Data (materials) associated with development of the intervention are suppled as Underlying data ^28^.

The method of implementation of the AIR intervention are yet to be validated. We describe further details of the intervention, steps of its pilot implementation and how we expect it to be implemented by the research community. We expect the method, after external peer review will provide a pathway for implementing family led home rehabilitation, especially in low resource settings.

### TIDieR Checklist Item 1: brief name of the intervention

The intervention was named AIR, an acronym for AIIMS ICU Rehabilitation.

### TIDieR Checklist Item 2: rationale, theory, or goal of the intervention

International efforts at tailoring healthcare to the unique needs of long-term tracheostomized patients with or without mechanical ventilation ^20, 23, 29, 30^ have involved multi-professional teams that coordinate regionally adapted training initiated at the tertiary-care centers and continued across healthcare sectors. Interpretation of the lived experiences of the five family patient dyads of the PRG revealed that the current care pathway for patients discharged home was disturbing for the patient and the family. Dyads reported physiological and emotional distress: “the hole in the neck is alarming” and “my neighbours will get scared when they see the hole”. Beyond the psychological and physical factors, we identified five sub-themes associated with the care pathway. These were, i, fear of caring for the airway and tracheostomy itself, ii, inability to ensure adequate nutritional intake for the patient, iii, concerns over costs and out-of-pocket expenditure related to hiring/purchase of equipment, iv, fear and helplessness while making unsupervised urgent decisions at home, v, insecurity over leaving the hospital sans a medical guardian. For healthcare providers, three themes emerged: i, lack of time for dedicated counselling or training of carers, ii, poor coordination of discharge pathways with clinical pressure for ICU beds versus logistic readiness for discharge, iii, doubts over carer ability to provide safe home-care within financial and logistic constraints.

These findings of the interpretative analysis were disseminated back to the CC and combined with a review of the literature to explore how home care/discharge packages could be used to inform the design of the AIR intervention. Themes i and ii (lack of confidence with care procedures) directly led to the development of the training intervention. The CC identified ten key components of daily care: catheter care, tracheostomy care, suctioning, monitoring oxygenation, home ventilation, enteral feeding, blood sugar management, bowel, and bladder care, handling common emergencies, and nutrition awareness.

Regarding theme iii (concerns over costs), it directly led to the decision to make available standard equipment and consumables, requisite information, and readily available troubleshooting (suction machines, oxygen concentrators, home ventilators, pulse oximeters, glucometers, hospital bed/backrest, airbed, suction catheters, gloves, gauze, betadine, tracheostomy tubes, indwelling catheters). Themes iv, v (fear and insecurity) led to a digital solution that would provide ongoing support and communication between the healthcare team and the patient/ family and post-discharge home visits and telephonic follow-ups. The CC recommended that the digital app would need to include training videos and curated ‘Frequently Asked Questions to provide an on-demand resource tool to support carers in the direct delivery of care at home. Suggestions for loading training videos into the application came from PRGs ‘ *training steps get forgotten with time, and videos or pictures will remind the carer’.* One PRG member suggested that building a community chat option in the mHealth application will enable sharing of stories among carers and provide psychological support. I&T expert, S&P personnel, PR, and CC discussions in the WG meetings codesigned the specifications for the mHealth app and agreed that a rental model of equipment procurement and handing over would best fit the requirements expressed by the PRGs ^31– 38^.

### TIDieR Checklist Item 4: procedures, activities, and processes used in the intervention

*An overview of the procedures and processes used in the intervention can be found in Figure 3.*

*Patient screening for inclusion.* Nurses in charge of clinical areas catering to tracheostomised patients (three ICUs, five high dependency units, and an emergency area) identified patients to a designated CC member. The RA screened patients. Eligibility for inclusion was - adult patients admitted to the ICU and tracheostomised, with a likelihood of surviving to hospital discharge, dependent on carers for activities of daily living such as enteral feeding, urinary catheter, bed, and bowel care. Those who were hemodynamically unstable, on high ventilatory, or requiring high oxygen support (>50%) at the assessment time were followed up every alternate day. Patients who had a tracheostomy solely for relieving airway obstruction and were otherwise independent were excluded. Patients who had no known relatives or if the relatives were incapacitated were excluded. The parent clinical team discussed the likelihood of survival beyond hospital discharge.

*Psychologist Counselling.* Once eligible, the psychologist (P) approached the patient and carer for consent and explained the aims of the AIR intervention using the support materials described in Checklist Item 3 below. Carers who were unsure were scheduled for a follow-up interview with a member of the CC and the treating physician. It was explained that the decision not to participate would not impact ongoing care. After consent, the Nurse Trainer (NT) assessed patient physiology for bedside training and began hands-on training using the materials described in item 3 of the TIDieR below.

*Carer training in tracheostomy management, nutrition, and personal care (prevention of pressure injury, catheter care, promotion of muscle/movement).* Each carer was explained the need for the procedures (training modules) and then demonstrated the correct steps. Subsequent sessions were a mix of hand holding and independent demonstration of the modules by the carers. The number of training sessions required depended on the carers' experience and attitude and could vary from a minimum of 2 to 6 sessions, each lasting for 30–45 minutes.

To assess training readiness, feedback from EBCD stages 2&3 above was used: the carer was asked to score his confidence subjectively before and after each module at each session on a numeric scale of 0–10, where 0 was ‘not confident at all’ and 10 was ‘very confident, can teach others’. When their scores reached 8 or more in a module, an objective assessment for technical performance was done by the NT on a score sheet developed by the CC. A score of > 8 on subjective confidence and >5 on objective performance was considered adequate to declare the carer ‘adequately trained’ in a module.

*Installation of the M-Health application.* The application was loaded on the carers’ mobile in the project room. They were trained in its use. The carers were encouraged to see the training videos, read the guides, and interact with the team to increase their confidence in post-discharge support.

*Equipment handover and set up.* One -two days before discharge, equipment vendors were invited to hand over the appropriate equipment and train the carers in the use and troubleshooting. A toolkit of daily-need consumables for 3–4 days was provided. The kit contains a head end-elevation support, an airbed, reusable gloves, suction catheters, 100ml saline bottles, gauze, chlorhexidine hand wash, an extra tracheostomy tube with heat-moisture filter, and an AMBU bag (purchased directly, not rental) was handed to the carer. The project covered equipment rental costs for one month for all patients: extendable at the discretion of the CC. The head end-elevation support, air bed, and AMBU bags were to be returned once the patient recovered or expired for cleaning and reuse.

*Home assessment and Exit Meeting* The project Field worker (FW) discussed the layout of the carers' home via photographs sent over the mobile. Patient placement after reaching home was agreed upon before discharge. An exit meeting with P and an RT member before discharge enabled carers to ask questions and the RT to get feedback and experiences (EBCD Stage 4).

*Home visit and follow-up.* Daily telephonic contact for the first three days by the NT was followed by FW-initiated follow-ups on days 7,14,21, and 28. The patient's quality of life and career burden was assessed with the EuroQOL 5D (EQ5D-3L) and Caregivers Burden Scale (CBS) on days 7 and 14. Nutrition status and risk assessment were done on all follow-ups by the patient-generated subjective global assessment short form (PG SGA) tool, 24-hour recall of dietary nutrition, and a survey to assess the barriers and facilitators of providing enteral nutrition to tracheostomy patients. Based on the feedback, a CC member and dietician-guided telephonic counselling was done for patients at high risk for malnutrition. Physical home visits began on days seven and 21 midways as an iterative modification based on feedback received in Carer Meeting 2. ( Figure 1)

Figure 3 details the procedures, activities, and processes used in the intervention

### TIDieR Checklist Item 3: physical or informational materials used in the intervention delivery or training

   A.  Physical or informational materials used to introduce and advertise the project:

- Poster: a one-page poster was designed to succinctly highlight the ‘Who’, ‘Why’, ‘Where’, and ‘How’ of the intervention. Benefits anticipated for the healthcare system and patient-family were mentioned. Along with the contact number of project personnel, the names of all core committee members, including intensivists, neurosurgeons, nursing, physiotherapy psychologist, and research assistant, were printed to enable easy reach out for patients and hospital personnel. This poster (English and Odia versions) was put up in various areas of the hospital (wards, casualty, and OPD). The goal was to get health care workers (HCWs), administrators, and patient-families curious. (Underlying data 1 ^28^)
- Brochure: a more detailed brochure in four pages (coloured, with representative photographs) was designed as patient-family handouts. The goal was to give the patient family a better understanding of its components and goals. (Underlying data 2 ^28^)
- Video: a video, 10 minutes in duration (in Odia, Bengali, and Hindi), was available to inform patient-family about the project.

   •   Consent form: the consent form had an information sheet attached to it.

   B.  Physical or informational materials used for training carers.

   •   Printed modules: step-by-step advice for caring for tracheostomy, oral and tracheostomy suctioning, bed care, nasogastric feeding, care of indwelling urinary catheter, monitoring blood sugar with a handheld device, and administering insulin, using a pulse oximeter, and bathing the patient was bundled into a booklet. The process was iterative, requiring three revisions that catered to carer suggestions, doubts, and queries before the final draft. (Underlying data 3 ^28^)

   •   Training videos: after collecting feedback from the first 25 patient-carer sets, we learned that the TT suctioning, nasogastric feeding, and bathing were most daunting for carers. With the feedback received, videos were prepared to show ‘real-world’ procedures being done, in one case by a real patient-carer (the patient bathing video).

   •   Assessment modules: to help the trainer assess the progress in training and increase self-confidence among the carers in patient care, a multidisciplinary team decided on a subjective self-assessment tool (Likert scale based) to be administered to the carer before and after each training session. A score sheet was created based on must-demonstrate, and good-to demonstrate skills for each module to help the trainer recognize the requirement for further training sessions. It was observed in the course of intervention design that some carers appeared very confident but were unable to demonstrate safe patient care adequately; therefore, two-pronged feedback was planned:

- Self-assessment tools that mapped the improvement in carer (trainee) self-confidence over each session and the entire training period.
- Objective assessment by the trainer of hands-on bedside patient care skills on the final day of training (when the carer self-confidence in each module is 8/10 or greater). (Underlying data 4 ^28^)

   •   Subjective global assessment of carer attitude, aptitude, and preparedness for home care by two faculty of the project team, not involved in training.

   •   Physical training in the use of equipment: hands-on training on equipment was provided to carers 1–2 days before planned patient discharge.

Dietary guide sheets: printed guides were given to the patients based on needs assessed by a dietician.

### TIDieR Checklist Item 5: description of the expertise, background, and speciﬁc training given to intervention providers

The CC conducted interview-based recruitment for the RA, the NT, FW, and the P. The RA was trained in biotechnology with prior experience in clinical work. The counselor was a trained psychologist, the NT a trained ICU nurse, and the FW trained pharmacist and paramedic. Each of these team members had more than two years of experience in their field and had successfully collaborated on similar service improvement projects in the healthcare system in the region. Further details of the roles appear in Figure 3. The CC and the RA conducted the departmental and project induction, including good clinical practice (GCP) training. The CC members ensured further integration into the hospital. Each had good spoken and written English, Odia, and Hindi.

The Equipment vendors and m-Health application experts were selected by an open call and meeting with the CC. Potential vendors were screened for track record and asked to tender quotations reviewed by the CC and assessed for adherence to the AIIMS service tender requirements.

*Training of Trainers.* The AIR training modules and printed materials were adapted from an already implemented curriculum delivered by the MoH, Government of India for home health aides ^39^.

The adaptations included simplifying language, using pictorial guides, and adding specific training in tracheostomy care and care of indwelling catheters. Modules underwent three iterations based on feedback from the project team. The NT and FW were trained in a nursing -simulation center by Critical care clinicians in the CC, with supervised bedside scenario training in each aspect of patient care, infection control practices, and objective assessment of carer training (using the 4-stage approach). Training for trainers was conducted in English (as this is the common language used for healthcare training in the region). All the project staff underwent additional training in troubleshooting standard equipment, installing, and loading the m-Health application, delivered by the respective working groups and overseen by the CC.

*Nutrition assessment and follow-up-* The first 25 assessment and advisory sessions were administered by a qualified dietician who worked with and understood the needs of tracheostomized ICU patients and families. Dietary suggestion charts were created for common scenarios. (Underlying data 5 ^28^) All project staff were trained in the use of the HealthifyMe mHealth app that caters to the local diet to quantify calories and macronutrient content of the diet consumed in the previous 24 hours (24 hr dietary recall)

*Data entry-* The research personnel were trained in eCRF data entry over a series of 3 sessions with online support from trainers from the CCAA. Study data were collected and managed using an E-CRF (REDCap electronic data capture tools) hosted at AIIMS Bhubaneswar (32).

### TIDieR Checklist Item 6: mode of delivery

The training was individually adapted and provided in groups of two participants supervised by the trainer. Some family members who had more significant difficulties in learning were invited to watch other families who had achieved greater confidence or were in more advanced training sessions.

The application was loaded on the mobile of carer in the project room. They were trained in its use. The family members were encouraged to see the training videos, read the guides and interact with the team prior to patient discharge to increase their confidence in post-discharge support.

The consumables and equipment were handed over two days predischarge. The family members were explained (and infographic given) that the equipment was NOT free: that they are being handed over as a gesture of support for a maximum of one month until patient recovery or death and for buying time for the family to procure them if needed longer. ID proof was retained until the equipment is returned to be used by another family.

### TIDieR Checklist Item 7: type(s) of location(s) where the intervention occurred, including any necessary infrastructure or relevant features

*Study Site-* AIIMS Bhubaneswar is a 900 bedded tertiary care teaching center having 100 adult ICU beds. The ICUs are managed by different departments, with most patients in this study being recruited from the Central ICU (14 beds, surgical & medical), Neuro-Respiratory (7 beds), Neurosurgery (7 beds), and Emergency Room (16 beds). The public-funded hospital is designated an Institute of National Importance, created to cater to the underprivileged population of the state of Odisha and neighboring regions. Admitting 40,000 inpatients annually, AIIMS conducts more than 15,000 surgeries requiring an inpatient stay. At any point, > 5 % of inpatients are extended-stay- patients, with a length of stay > 21 days. These patients most commonly belong to neurosurgery, neurology, general medicine, and emergency-trauma departments. Induction and obtaining consent were done in face-to-face meetings. Although an attempt was made to have all the meetings in a designated space—the ‘project room’, the research assistant and psychologist were free to choose any quiet space where speaking to family members would be convenientTraining sessions were always given at the patient's bedside after confirming with the treating nurse.

Exit interviews with ICU faculty were in a designated office room with facilities for audio-visual display; persistent questions and remaining fears were addressed.

Follow up at days 14 and 21 are telephonic, on video calls, or at hospital visits (if the patient had returned for assessment with the parent department).

Home visits are made on days 7 and 28 after discharge.

### TIDieR Checklist Item 8: number of times the intervention was delivered and over what period of time including the number of sessions, their schedule, and their duration, intensity or dose

AIR delivered from patient identification and consent to one month after discharge from the hospital. Contact with the patients and family carers continues until patient death or recovery as they retain access to the mobile application and protect the team's contact details.

The initial scouting was rapid, taking about 10 minutes, followed by an assessment and counselling session that lasted approximately 45 minutes.

Bedside training sessions took between 45 minutes to one hour for the first session; subsequent sessions took between 30 to 45 minutes each. A minimum of three sessions are provided;sessions were given until the carers’ subjective confidence in each module improves to >8 and objective scores given by the trainer are >5.

The field worker wasexpected to spend at least 2 hours with the patient, retraining the carers at the home visits on days 7 and 21.

The psychologist spent 10–20 minutes in the follow-up calls scheduled on days 1–3, 14, and 28.

### TIDieR Checklist Item 9: tailoring of the intervention

The content has been closely developed alongside a core team consisting of medical, paramedical, non-medical, and laypersons. It has been informed by research identifying the experiences, distress, needs, and preferences for support families looking after chronically ill patients with tracheostomies *in situ* ^6, 8, 27, 29, 30, 38– 54^.

Examples of tailoring for the population include:

- The use of participatory action research methodology: face-to-face workshops with a total of five relatives (one husband, three sons, and one wife) of tracheostomized patients previously rehabilitated home and were involved as family-carer research partners. One clinical psychologist was involved as an expert research partner. Research partners and research group members worked collaboratively throughout the study. Data were analysed iteratively using written summaries of the workshops parallel to data collection.
- Demonstrating videos of previously rehabilitated patients and enabling contact with them *via* telephone or community chat option in the mobile application for closer community networking.
- The inclusion of counselling in the context of the situation of being a family member of a chronically ill patient ( *e.g.*, fear of sudden complication).
- The choice between attending the initial assessment and counselling sessions *via* telephone, face to face at bedside/project room or cafeteria.
- All dietary and nutrition advice was tailored to local Odia cuisine segregated based on seasonal availability and cost.

### TIDieR Checklist Item 10: modiﬁcations of the intervention during the study

As the intervention was being developed iteratively, adaptations, titrations, and personalisation were an ongoing process. To date, the adaptions introduced are presented in Table 1. 

**Table 1.** TIDieR Item 10. Modifications of the intervention.

S No		Previously	After Iteration	Reason
1.	Inclusion Criteria	i. Expected to survive to discharge and to need tracheostomy beyond ICU discharge. Hemodynamically stable, on weaning mode of ventilation. ii. Within a radius of 200 km	All tracheostomy patient‘ ‘are expected to survi’e’ even those currently on the control mode of ventilator support and vasopressors. Any patient-carer dyad accepting the intervention (equipment transport rates levied beyond 200km)	To allow early consenting and training of carers, thereby avoiding premature home discharge due to the pressure on ICU and hospital beds. Worries about equipment safety and retrieval subsided, and vendors agreed to new terms of supply.
2.	Hands-on training of carer	One-on-one training. One family at a time. Minimum 3 sessions.	Carers with significant difficulties in learning were invited to watch ongoing sessions of more confident carers or those in more advanced stages.	Peer learning and watching other carers perform was an incentive that improved acceptance.
3	Videos of common procedures	The first iteration had videos filmed in simulated settings	Suctioning, Nasogastric feeding, and bathing videos were remade, in a real-world setting	Carer feedback suggested greater satisfaction with real-world videos
4	Project Related Infographics‘	‘Free access to equipment bank for patient care’	Availability of equipment and consumables as a part of the project was downplayed, and a rider added’– ’ *If* the carers pass knowledge-test and demonstrate the ability to perform basic care-giving tasks, *then * *the* equipment will be provided free of cos’.‘	‘Free equipment’ without adequate training in care and use of equipment led to few demands of equipment as ‘ ‘right’ without devoting time to training.
5	Exclusion Criteria	i. Bedbound, but decannulated predischarge were excluded	Patients needing enteral feeding/ catheter care were followed up as a part of the project.	The rapport built with the carers continued post-discharge
		ii. Carers not trained adequately due to late consent or conflicted carer situation	ii. Carers willing to use the mobile app and stay in touch were included.	Conflicts in or changed final-carer status were not uncommon: all stakeholders agreed that the other three limbs of the app would help home rehabilitation.
6	Eligibility for free equipment	Patients and carers below the poverty line (having ‘ ‘B’L’ card ) could avail the free Equipment bank	A one-month free facility for all patients: followed by a consideration based on a case-to-case basis. The family members were explained (and infographic given) that the equipment is NOT free: they are being handed over as a gesture of support for a maximum of two months until patient recovery or death and buying time for the family to procure them if needed longer.	Providing equipment free initially improved acceptance and

### TIDieR Checklist Item 11: planned procedures for how adherence or fidelity was assessed, describe how and by whom, and if any strategies were used to maintain or improve fidelity, describe them

We will use a mixed methods approach to assess the fidelity, feasibility, appropriateness, and acceptance of the pilot AIR intervention by the stakeholders. The Acceptability of Intervention Measure (AIM), Intervention Appropriateness Measure (IAM), & Feasibility of Intervention Measure FIM, AIM, IAM framework will be used to evaluate stakeholder perspectives regarding the feasibility, acceptability, and appropriateness of the intervention ^55^. This evaluation tool was chosen for its simplicity of assessment and ability to be conducted via telephone. Essential elements for fidelity are predefined (the proportion of approached patient-carer dyads counseled, consented, and received the components of the intervention). Data are kept on the number of patient-carers approached, proportion consented, and proportion completed each intervention step.

A mixed methods process evaluation will include assessment of the quantitate training assessments, data on fidelity of the intervention, analysis of case mix, and outcomes for patients included and not included in the project from the existing IRIS registry. In addition, semi-structured interviews with carers and providers will be analyzed and triangulated with the feedback during stages 3 and 4 of the EBCD to explore stakeholder perspectives regarding the project and to identify barriers and facilitators to implementation prior to scaling up ^56^.

### TIDieR Checklist Item 12: actual adherence or fidelity

Actual adherence to AIR will be reported in the results of the ongoing single-arm feasibility study registered prospectively in the Clinical Trials Registry India ( CTRI/2020/11/029443 [Registered on: 27/11/2020] Trial Registered Prospectively).

## Discussion

Patients with long-term ICU stays requiring tracheostomies and their carers have complex needs, with international evidence indicating poor outcomes and quality of care ^57^. We describe the development and details of a multicomponent intervention to facilitate carer-led home discharge for tracheostomy patients.

*Using a stakeholder-led- codesign approach.* Participatory approaches to the design and implementation of services for healthcare are relatively new internationally but remain uncommon in LMICs. Perceptions regarding their selection and use may include concerns regarding the time and costs of engagement, the underpinning knowledge and expertise available within the patient and public stakeholders, and for researchers and health care providers to apply these approaches to research ^58, 59^. The RT in the AIR project selected the EBCD approach as participatory, stakeholder-led development, and implementation of the intervention appeared essential to successful delivery.

We found that carers (predominantly female, elderly, or less educated) had difficulty recognizing home-rehabilitation as a process: accepting a top-down approach rather than suggesting changes and following the orders of health care teams came more naturally. Triggers such as analogies, showing pre-recorded videos of previous carers relating their experiences, and one-on-one meetings with CC members or project staff led to richer discussions than meetings with more stakeholders. Scheduling meetings at a time and place of choice of the stakeholder- whether patient-carer or WGs or other HCWs worked best. Greater carer focus on ‘free equipment’ compared to getting trained had to be circumvented with a conditional approach: the equipment would be given only when patient–care skills are demonstrated. The one-month rental policy with keeping an identity document as collateral to ensure safe-keep and return was a suggestion of PRGs. There was a reluctance among carers to initiate success sharing over the community chat section in the mHealth app because of a superstition of ‘the evil- eye’ among some carers. Nevertheless, we felt the benefits of codesign were substantial. Modifications and adaptations shaped contextually and situationally were informed by feedback from carers, program staff, treating physicians, and nursing staff caring for chronically ill tracheostomized patients in the ICUs.

*Barriers to implementation:* Barriers to home care programs in the literature include an absence of adequate hands-on training ^15^, discharge planning with systematized follow-up, logistic inadequacy (literacy, water, electricity), and suboptimal community support ^60, 61^. Stakeholder perspectives have focused more on the patient-carers, who report feeling scared, helpless, judged, and isolated on their home-rehabilitation journeys ^61^. Other than those reported, the COVID-19 pandemic introduced unique challenges for us. Family hierarchy, gender, and scheduling issues had to be circumvented or worked into all the stages of development and iterative modifications. The iterations and tailoring described in sections 9 and 10 of the TIDieR described how we identified and overcame barriers to implementing AIR.

*Using the TIDieR to describe AIR-* The TIDieR checklist uses a clear structure and includes items relevant to a home rehabilitation intervention (including procedures, training materials, and mode of delivery). However, we faced some difficulty in describing specific interrelated components sequentially under the checklist provided, and subcategories were needed in some places to describe the intervention processes.

In line with the TIDieR checklist, the detailed description of AIR we hope will help facilitate fidelity to the protocol during the implementation and evaluation. Furthermore, clinical delivery will be replicable if AIR is scaled up later.

### Strengths and limitations

The AIR intervention was developed incorporating invaluable feedback from sll stakeholders and further adapted to enhance the acceptability of the local population. Adopting a more structured framework to inform the cultural adaptation of evidence-based interventions may improve acceptability and relevance.

## Conclusions

We detail the development of the AIR intervention and describe it in detail, as informed by phase I of the MRC guidance ^62^. The provision of structured intervention protocols in sufficient detail aids the implementation of complex interventions and reduces research waste ^63^, providing tools to maximize protocol fidelity ^56^.

## Data Availability

Figshare: AIIMS ICU Rehabilitation (AIR): healthcare intervention development and description. Underlying data. https://doi.org/10.6084/m9.figshare.23445329 ^28^. This project contains the following extended data: Poster / Infographic for the intervention (for healthcare workers) Brochure for patients and carers in local language Training module for trainers and carers Tool for Assessment of Carer proficiency Diet charts in local language. Data are available under the terms of the Creative Commons Zero "No rights reserved" data waiver (CC0 1.0 Public domain dedication).
