# Supplementary material for: AIIMS ICU Rehabilitation (AIR): development and description of intervention for home rehabilitation of chronically ill tracheostomized patients
Source: Wellcome Open Res. 2025 Feb 28;8:285. Originally published 2023 Jun 30. [Version 4] doi: 10.12688/wellcomeopenres.19340.4 (PMC11399758; doi:10.12688/wellcomeopenres.19340.4)
Supplement: Peer review history

# Reviewer report: Reviewer response for version 3

**DOI:** 10.21956/wellcomeopenres.25280.r104190

**Published:** 2024-11-04

**Authors:** Lloyd Bradley

**Affiliations:** 1 Royal Hospital for Neurodisability, London, UK

This is an interesting and important intervention, the development of which has clearly involved a lot of work. The study encompasses a lot of different methodologies and approaches and some of these could, perhaps, be more well described. There are a few statements / terms which require references and some minor textual issues but these are easily addressed.

 Specific comments;

 Page 1

- Is “chronically ill” ever defined? Can one have a tracheostomy and be well? Although I appreciate this phrase is used in the title and throughout the manuscript might it not be more appropriate to frame this as “patients discharged from hospital with long term tracheostomies” particularly as the diagnoses of the patient group are never defined or specified.

Page 2

- What is “Patients-carer”? Does this refer to patients and carers?

Page 4

- “referred to as the chronic critically ill (CCI)” – is there a reference for this statement
- “Prolonged need for mechanical ventilation due to cardiopulmonary disease, inability to protect the airway due to neurological dysfunction or brain injury are the main reasons for tracheostomy in the CCI.” – again, needs a reference
- “The implications and outcomes (including cost) following protracted ICU stay are potentially devastating for patients and their families. Reasons for this include limited availability of and access to rehabilitation services, lower baseline health literacy, concomitant infections in the presence of antibiotic resistance, and spiralling financial burden associated with healthcare costs and loss of income” – this needs rewriting so that it refers to specifically LMICs, antibiotic resistance is not really relevant and it would be worth emphasising the loss of income of family members needing to adopt caring roles.
- “Causes put forth” – suggest add a word to make; “causes for lack of successful implementation put forth”
- “In many instances” – do you mean in many current instances (of other models)?

Page 6

- “ST,JM,BR,UH, all intensive care physicians, noting repeated readmissions of tracheostomised patients discharged from the ICU due to hospital acquired infections, conducted an audit of hospital length of stay of inpatients. The results demonstrated unusually protracted lengths of stay for adult tracheostomised in comparison to non-tracheostomised patients” – could you clarify this? Does this mean re-admissions to the ITU from general wards (Hospital acquired infections)? Or from home? Does admission length refer to re-admissions or admissions in general? There is no further reference to hospital or ICU re-admission (although this would be a good outcome measure

Page 7

- There are different qualitative methodologies referred to e.g “realist review”, “interpretative analysis”; these require references so that readers can understand what they are
- If I have read the methods correctly, did the authors undertake a systematic review (albeit realist)?  - this requires more description of search terms / strategies and results, as it is this appears not be properly described or explained

Page 8

- I’m not sure that “stakeholder engagement” in the sense of co-design or dissemination is really whats being described here. Suggest reviewing this as congratulating participants isn’t the same as engaging them.

Page 9

- As above “Findings from literature review and codesign exercises” needs more explanation

Page 10

- “each carer was explained the need for the procedures (training modules) and then
- demonstrated the correct steps” – suggest change to “each carer was educated in the need…. And then shown the correct steps”
- “to score his confidence” – suggest change to their confidence

Page 11

- Training rather than trainings
- On the mHealth diagram, discharge advice rather than advise

Page 14

- “of a superstition of ‘the evil- eye’ among some carers” – could this be described or some sort of reference given?

Page 15

- The conclusion could be a bit stronger / more positive. Developing a novel tool for this challenging issue which could be utilised much more widely is worth stating with more confidence!

Is the rationale for developing the new method (or application) clearly explained?

Yes

Is the description of the method technically sound?

Yes

Are the conclusions about the method and its performance adequately supported by the findings presented in the article?

Yes

If any results are presented, are all the source data underlying the results available to ensure full reproducibility?

Yes

Are sufficient details provided to allow replication of the method development and its use by others?

Yes

Reviewer Expertise:

Rehabilitation processes and delivery

I confirm that I have read this submission and believe that I have an appropriate level of expertise to confirm that it is of an acceptable scientific standard, however I have significant reservations, as outlined above.

# Response

**Published:** 2025-01-23

**Authors:** Swagata Tripathy

**Affiliations:** Anesthesia and Critical Care, All India Institute of Medical Sciences, Bhubaneswar, Odisha, India

We thank the reviewer for their valuable insights and comments. A point to point response follows, with a edits incorporated in the revised  manuscript  Page 1

 •    Is “chronically ill” ever defined? Can one have a tracheostomy and be well? 

***Response: **I understand the reviewers concern. Our team has debated with this concept, in an effort to identify the most appropriate term.*

*There is a fairly large subset of patients who get tracheostomies for airway obstruction, post-surgery for cancer etc, who are mobile and not bed ridden. Therefore, we have carefully chosen this terminology to better describe the population which we are targeting.* *As mentioned in the manuscript, this is an accepted definition- ". These patients who have survived a period of acute illness but are not yet free from life-sustaining therapies are referred to as chronic critically ill (CCI).*

*The consensus definition for CCI combines two factors: care received in the ICU for at least eight days and at least one out of five eligible conditions (prolonged mechanical ventilation (MV); tracheostomy; sepsis; severe wounds and/or multiple organ failure; ischemic stroke, intracerebral haemorrhage or traumatic brain injury) 2. Prolonged need for mechanical ventilation due to cardiopulmonary disease and inability to protect the airway due to neurological dysfunction or brain injury are the main reasons for tracheostomy in the CC"*

 Although I appreciate this phrase is used in the title and throughout the manuscript might it not be more appropriate to frame this as “patients discharged from hospital with long term tracheostomies” particularly as the diagnoses of the patient group are never defined or specified.

***Response: **We hope the reference provided and the definition will describe our cohort. See response above.*

 Page 2

 •    What is “Patients-carer”? Does this refer to patients and carers?

***Response: **Yes, the dyad of the patient and their carer as a composite has been referred to as a patient-carer.*

 Page 4

 •    “referred to as the chronic critically ill (CCI)” – is there a reference for this statement ***Response: **Consensus definition for CCI combines two factors: care received in the ICU for at least eight days, and at least one out of five eligible conditions (prolonged mechanical ventilation (MV); tracheostomy; sepsis; severe wounds and/or multiple organ failure; ischemic stroke, intracerebral haemorrhage or traumatic brain injury) 2 .*

*Reference- Number 2- Carson SS: Conference Proceedings Definitions and Epidemiology of the Chronically Critically Ill. Introduction Definitions of Chronic Critical Illness Defining CCI by Tracheostomy Prolonged Mechanical Ventilation Long-Term Mechanical Ventilation Composite Definitions Epidemiology of Chronic Critical Illness Outcomes Summary.2012.* *•    “Prolonged need for mechanical ventilation due to cardiopulmonary disease, inability to protect the airway due to neurological dysfunction or brain injury are the main reasons for tracheostomy in the CCI.” – again, needs a reference*

*Thank you for pointing out that the refernce is missing here. The reference is the same as the preceding sentence. We have now mentioned this in the manuscript.*

 •    “The implications and outcomes (including cost) following protracted ICU stay are potentially devastating for patients and their families. Reasons for this include limited availability of and access to rehabilitation services, lower baseline health literacy, concomitant infections in the presence of antibiotic resistance, and spiralling financial burden associated with healthcare costs and loss of income” – this needs rewriting so that it refers to specifically LMICs, antibiotic resistance is not really relevant and it would be worth emphasising the loss of income of family members needing to adopt caring roles.

***Response: ** We appreciate the suggestion. The statement has been modified to refer specifically to LMICs.*

 •    “Causes put forth” – suggest add a word to make; “causes for lack of successful implementation put forth”

***Response: **Revised as suggested.*

 •    “In many instances” – do you mean in many current instances (of other models)?

***Response: **We meant  ‘In many instances (of published reports)’. This has now been added.*

 Page 6

 •    “ST,JM,BR,UH, all intensive care physicians, noting repeated readmissions of tracheostomised patients discharged from the ICU due to hospital acquired infections, conducted an audit of hospital length of stay of inpatients. The results demonstrated unusually protracted lengths of stay for adult tracheostomised in comparison to non-tracheostomised patients” – could you clarify this? Does this mean re-admissions to the ITU from general wards (Hospital acquired infections)? Or from home? Does admission length refer to re-admissions or admissions in general? There is no further reference to hospital or ICU re-admission (although this would be a good outcome measure

***Response: **We have now rewritten the paragraph to clarify it. “. ST,JM,BR,UH, all intensive care physicians, noting repeated readmissions of tracheostomised patients discharged from the ICU (from the hospital wards after step down) due to hospital acquired infections, conducted an audit of hospital length of stay of inpatients. The results demonstrated unusually protracted lengths of stay for adult tracheostomised with greater readmissions to the ICUs in comparison to non-tracheostomised patients”*

*Although we have not referenced ICU/ hospital readmissions further, the LOGIC diagram and intervention programme theory focused to reduced length of stay and and decreased nosocomial infections, which we considered a representative measure in the intervention development stage. As correctly pointed out, ICU/ hospital readmissions is a good outcome measure and we are measuring it presently in the ongoing Intervention Effectiveness Evaluation phase.*

 Page 7

 •    There are different qualitative methodologies referred to e.g “realist review”, “interpretative analysis”; these require references so that readers can understand what they are. 

*References have been added as suggested.* •    If I have read the methods correctly, did the authors undertake a systematic review (albeit realist)?  - this requires more description of search terms / strategies and results, as it is this appears not be properly described or explained.

***Response: **The realist review was an extensive undertaking and is in the process of manuscript preparation presently. Detailed description will be beyond the purview of this manuscript. However,   the methodology, databases referenced and search terms appear in the review protocol, for which we have provided the details so the interested reader may refer further.*

*PROSPERO registration ID- CRD42022347389*

 Page 8

 •    I’m not sure that “stakeholder engagement” in the sense of co-design or dissemination is really whats being described here. Suggest reviewing this as congratulating participants isn’t the same as engaging them.

***Response: **We wish to humbly differ here , and point out that stakeholder engagement refers to a broad variety of activities, although codesign and dissemination are among them.*

*“Stakeholder engagement can be defined as practices that the organization undertakes to involve stakeholders in organizational projects or activities (Greenwood 2007).”*

*We believe the exercises mentioned truly encouraged cross engagement among all stakeholders and not only congratulated them.*

 Page 9

 •    As above “Findings from literature review and codesign exercises” needs more explanation.

***Response: **Expanded.*

 Page 10

 •    “each carer was explained the need for the procedures (training modules) and then

 •    demonstrated the correct steps” – suggest change to “each carer was educated in the need…. And then shown the correct steps”

 •    “to score his confidence” – suggest change to their confidence

***Response: **Both suggested modifications are incorporated.* Page 11

 •    Training rather than trainings

***Response: *** Revised as suggested.

 •    On the mHealth diagram, discharge advice rather than advise

***Response: *** Corrected Page 14

 •    “of a superstition of ‘the evil- eye’ among some carers” – could this be described or some sort of reference given?

***Response: *** Added

 Page 15

 •    The conclusion could be a bit stronger / more positive. Developing a novel tool for this challenging issue which could be utilised much more widely is worth stating with more confidence!

***Response: *** Thank you, we have now revised the conclusion .

# Reviewer report: Reviewer response for version 3

**DOI:** 10.21956/wellcomeopenres.25280.r97607

**Published:** 2024-09-13

**Authors:** Andrew Bates

**Affiliations:** 1 University of Southampton, Southampton, England, UK

Thank you for responding positively to my suggestions. There remains one outstanding issue.

 The full paper discussion does not reflect the changes you've suggested in your response. Please could you doublecheck and make sure to include these revisions. The discussion needs to include in its mature form the air intervention **hopes** to engage stakeholders....'

 I wish you the best of luck with your indexing and your project, it looks very worthwhile.

Is the rationale for developing the new method (or application) clearly explained?

Yes

Is the description of the method technically sound?

Yes

Are the conclusions about the method and its performance adequately supported by the findings presented in the article?

Partly

If any results are presented, are all the source data underlying the results available to ensure full reproducibility?

No source data required

Are sufficient details provided to allow replication of the method development and its use by others?

Yes

Reviewer Expertise:

Critical care recovery.

I confirm that I have read this submission and believe that I have an appropriate level of expertise to confirm that it is of an acceptable scientific standard.

# Reviewer report: Reviewer response for version 2

**DOI:** 10.21956/wellcomeopenres.22855.r83838

**Published:** 2024-06-08

**Authors:** Andrew Bates

**Affiliations:** 1 University of Southampton, Southampton, England, UK

Thank you for inviting me to review this manuscript. 

 The authors report the method for developing a home rehabilitation program for tracheostomised patients.

 The description of the methodology is thorough and detailed. 

 I can see that the authors have used the TIDIER intervention description checklist, however, I would like to see a more detailed description of the M-Health application. The other components are adequately described and would enable replication. 

 Authors do not report results, which will follow in a separate feasibility and process evaluation report. However, the first paragraph of the discussion refers to increased carer confidence, facilitated home discharge and continuation of support. These statements are not supported by results in the manuscript and should be moderated.

 The plan for future evaluation is appropriate to the intervention and consistent with MRC guidance for evaluation of complex healthcare interventions. I look forward to reading the results paper.

 Overall, the authors should be applauded for a thorough and well written report of the methods used to develop a complex healthcare intervention. With the two minor revisions requested above this manuscript will help researchers and clinicians to replicate the development of such an intervention.

Is the rationale for developing the new method (or application) clearly explained?

Yes

Is the description of the method technically sound?

Yes

Are the conclusions about the method and its performance adequately supported by the findings presented in the article?

Partly

If any results are presented, are all the source data underlying the results available to ensure full reproducibility?

No source data required

Are sufficient details provided to allow replication of the method development and its use by others?

Yes

Reviewer Expertise:

Critical care recovery.

I confirm that I have read this submission and believe that I have an appropriate level of expertise to confirm that it is of an acceptable scientific standard, however I have significant reservations, as outlined above.

# Response

**Published:** 2024-08-09

**Authors:** Swagata Tripathy

**Affiliations:** Anesthesia and Critical Care, All India Institute of Medical Sciences, Bhubaneswar, Odisha, India

Dear Dr Bates,

 I and my team and supervisors thank you for the time you spent to go through the manuscript and the useful suggestions to improve it further. I hope the changes/ additions made in response are satisfactory.

 Warm Regards,

 Swagata Tripathy,

 MD, DNB, IDCC, EDIC, FSNCC( Hon), FICCM

 Fellow Neuroanesthesia, Walton Centre, Liverpool

 Vice President Society of NeuroCritical Care India

 Professor & HOD

 Department of Anesthesia & Critical Care

 AIIMS Bhubaneswar.

**Point to point response-**

 Thank you for inviting me to review this manuscript. The authors report the method for developing a home rehabilitation program for tracheostomised patients. The description of the methodology is thorough and detailed.

***Answer: **Thank you for the kind words. We appreciate the time taken by you to help us improve the manuscript,  and for the suggestions .*

 I can see that the authors have used the TIDIER intervention description checklist, however, I would like to see a more detailed description of the M-Health application. The other components are adequately described and would enable replication. 

***Answer: **We appreciate the interest in the details of the application. In view of the word limit we have added a description and a figure to explain it in greater detail.*

 Authors do not report results, which will follow in a separate feasibility and process evaluation report. However, the first paragraph of the discussion refers to increased carer confidence, facilitated home discharge and continuation of support. These statements are not supported by results in the manuscript and should be moderated.

***Answer: **The observation is correct, thank you for pointing this out. The statements  have now been moderated to-*

 "In its mature form, the AIR intervention engages stakeholders from the initiation of patient recruitment to home discharge and follow up in a way that hopes to empower families to care for their tracheostomised bed ridden patients at home, by using the four components of the intervention: the hands-on training for carer confidence, the equipment bank allowing for facilitated home discharge in our economically challenged population, the mHealth app allowing bidirectional communication and home visits for continuation of support."

 The plan for future evaluation is appropriate to the intervention and consistent with MRC guidance for evaluation of complex healthcare interventions. I look forward to reading the results paper.

***Answer: **Grateful for your comment. We will love for you to review and guide our evaluation manuscript, if possible.*

# Reviewer report: Reviewer response for version 2

**DOI:** 10.21956/wellcomeopenres.22855.r77720

**Published:** 2024-04-13

**Authors:** Alison M Mudge

**Affiliations:** 1 Faculty of Medicine, The University of Queensland, Herston, Queensland, Australia

The manuscript is much improved and I amend my previous assessment to approved. 

 I noted just two typographical errors which you may choose to amend:

1. Three sentences are inadvertently repeated on end page 10/beginning page 11 (subsection "intervention bundles/The M-Health application")
2. In the last cell of table 1 (line 6) there is an incomplete sentence

Is the rationale for developing the new method (or application) clearly explained?

Yes

Is the description of the method technically sound?

Yes

Are the conclusions about the method and its performance adequately supported by the findings presented in the article?

Partly

If any results are presented, are all the source data underlying the results available to ensure full reproducibility?

Partly

Are sufficient details provided to allow replication of the method development and its use by others?

Partly

Reviewer Expertise:

Health services research, implementation of complex healthcare interventions

I confirm that I have read this submission and believe that I have an appropriate level of expertise to confirm that it is of an acceptable scientific standard.

# Reviewer report: Reviewer response for version 1

**DOI:** 10.21956/wellcomeopenres.21425.r68014

**Published:** 2023-11-16

**Authors:** Alison M Mudge

**Affiliations:** 1 Faculty of Medicine, The University of Queensland, Herston, Queensland, Australia

Re: AIIMS ICU Rehabilitation (AIR): development and description of intervention for home rehabilitation of chronically ill tracheostomized patients using the TIDieR checklist

 The authors are to be commended for describing a complex intervention designed for a low research setting which included engagement of patient carers through a partnership approach. I appreciate the importance but also the difficulty of reporting this development work.

 The authors set a clear introduction to the work, describing the need and importance. However I found the reporting confusing in places which might be improved by considering the following comments:

1. It is not always clear whether the authors are reporting the overall design work, the stakeholder engagement process, or the reporting process. For example the methods section refers to ‘mixed methods’ which appears however to refer to literature sought/used in design, and then mostly describes the participatory design process; the results describe the co-design process and the interventions components; the discussion briefly attends to observations about the co-design process but also the challenges of using the reporting checklist. I think part of the challenge is trying to use the TIDIER checklist to structure a results section which is problematic for narrative flow as it includes items from background, methods and results; more commonly a reporting checklist for any study type would be submitted as a supplementary file with the page reference for where those data are reported, as an aid to ensuring complete reporting.
2. I note reference to the MRC guidance for designing complex interventions. I suggest that the authors might find O’Cathain’s specific guidance on developing complex interventions ^1^ a more suitable structure for reporting this work, perhaps using the ‘actions to consider’ and potential outputs to guide how the current report is structured, and then supplying the TIRIER checklist as supplementary material. It might help resolve some of the confusing jumping around (eg reporting checklist item 4 before 3) and the repetition that exists currently between reporting the codesign actions and their findings and then subsequent refinement. A logic model would be a useful addition to tie theory/literature (mentioned briefly at the start of methods but not further articulated) and some of the co-design findings to planned activities and intended outcomes, which is still not well articulated and will be important for informing evaluation of the process
3. It would be helpful if the final bundle of four interventions (what each looks like, how it Is delivered, who is eligible for them)—could be more clearly articulated in a single part of the results.
4. The partnership process in this manuscript is variously called experience-based co-design, participatory action research, participatory design and co-design throughout the article. The actual activities of the PRG are not clear to me form text and figure 1 and I am not clear whether the boxes marked ‘carer meet’ refer to the PRG or ad hoc meetings with carers who were trialling materials. The methods do not fully adhere to EBCD methods, as there is not a clear description of in situ observation of practices nor use of a ‘trigger’ film highlighting the touchpoints that was used in a combined meeting of patients/carers and providers—carer videos instead are described as part of educational resources. I suggest use of a less specific term for the co-design method throughout the manuscript.
5. I found it difficult to follow in the results at times what were iterative design steps and what involved actual pilot of the processes and tools in the clinical setting (as there appear to have already been 200 patients enrolled in this new process). This was complicated by inconsistent use of past, present and future tense in the results section.
6. I suggest that research activities (eg planned feasibility study mentioned under checklist item 11 and research data collection mentioned under item 5) are separated clearly from the intervention design in the results and discussion.
7. I suggest reframing the discussion to briefly describe the final intervention and how it addresses the problem; compare with other approaches in the literature; discuss the strengths and weaknesses of the development approach, and particularly how generalisable the intervention might be or what local adaptation would be required for others to trial it; and outline plans for locally evaluating the intervention now that it is mature.

Is the rationale for developing the new method (or application) clearly explained?

Yes

Is the description of the method technically sound?

Yes

Are the conclusions about the method and its performance adequately supported by the findings presented in the article?

Partly

If any results are presented, are all the source data underlying the results available to ensure full reproducibility?

Partly

Are sufficient details provided to allow replication of the method development and its use by others?

Partly

Reviewer Expertise:

Health services research, implementation of complex healthcare interventions

I confirm that I have read this submission and believe that I have an appropriate level of expertise to confirm that it is of an acceptable scientific standard, however I have significant reservations, as outlined above.

## References

[1] : Guidance on how to develop complex interventions to improve health and healthcare. *BMJ Open* .2019;9(8) : 10.1136/bmjopen-2019-029954 e029954 10.1136/bmjopen-2019-029954 31420394 PMC6701588

# Response

**Published:** 2023-11-30

**Authors:** Swagata Tripathy

**Affiliations:** Anesthesia and Critical Care, All India Institute of Medical Sciences, Bhubaneswar, Odisha, India

Re: AIIMS ICU Rehabilitation (AIR): development and description of intervention for home rehabilitation of chronically ill tracheostomized patients using the TIDieR checklist

 The authors are to be commended for describing a complex intervention designed for a low research setting which included engagement of patient carers through a partnership approach. I appreciate the importance but also the difficulty of reporting this development work. **Thank you for the comment.**

 The authors set a clear introduction to the work, describing the need and importance. However I found the reporting confusing in places which might be improved by considering the following comments:

1. It is not always clear whether the authors are reporting the overall design work, the stakeholder engagement process, or the reporting process. For example the methods section refers to ‘mixed methods’ which appears however to refer to literature sought/used in design, and then mostly describes the participatory design process; the results describe the co-design process and the interventions components; the discussion briefly attends to observations about the co-design process but also the challenges of using the reporting checklist. I think part of the challenge is trying to use the TIDIER checklist to structure a results section which is problematic for narrative flow as it includes items from background, methods and results; more commonly a reporting checklist for any study type would be submitted as a supplementary file with the page reference for where those data are reported, as an aid to ensuring complete reporting.

**We appreciate the observation by the reviewer, and thank her for the suggestion to use the structure suggested by O’ Cathain et al to report the development and description, keeping the Tidier as a supplement. We have restructured the manuscript accordingly.**

1. I note reference to the MRC guidance for designing complex interventions. I suggest that the authors might find O’Cathain’s specific guidance on developing complex interventions 1 a more suitable structure for reporting this work, perhaps using the ‘actions to consider’ and potential outputs to guide how the current report is structured, and then supplying the TIRIER checklist as supplementary material. It might help resolve some of the confusing jumping around (eg reporting checklist item 4 before 3) and the repetition that exists currently between reporting the codesign actions and their findings and then subsequent refinement. A logic model would be a useful addition to tie theory/literature (mentioned briefly at the start of methods but not further articulated) and some of the co-design findings to planned activities and intended outcomes, which is still not well articulated and will be important for informing evaluation of the process

**We have now  edited the methods and results into ‘actions’ and ‘outcomes’ framework as suggested. A logic model has also been added now (Figure 3).**

1. It would be helpful if the final bundle of four interventions (what each looks like, how it Is delivered, who is eligible for them)—could be more clearly articulated in a single part of the results.

**We have now created a separate section titled ‘The AIR intervention’ which describes the four intervention components separately in greater detail.**

1. The partnership process in this manuscript is variously called experience-based co-design, participatory action research, participatory design and co-design throughout the article. The actual activities of the PRG are not clear to me form text and figure 1 and I am not clear whether the boxes marked ‘carer meet’ refer to the PRG or ad hoc meetings with carers who were trialling materials. The methods do not fully adhere to EBCD methods, as there is not a clear description of in situ observation of practices nor use of a ‘trigger’ film highlighting the touchpoints that was used in a combined meeting of patients/carers and providers—carer videos instead are described as part of educational resources. I suggest use of a less specific term for the co-design method throughout the manuscript.

**We agree that different terms have been used to describe the partnership process, and this might be a result of the difference in definitions given variously in published articles. However, we are happy to accept the reviewer’s suggestion, and have now simplified this by using the term- ‘codesign’ to refer to the partnership process throughout. **

1. I found it difficult to follow in the results at times what were iterative design steps and what involved actual pilot of the processes and tools in the clinical setting (as there appear to have already been 200 patients enrolled in this new process). This was complicated by inconsistent use of past, present and future tense in the results section.

**We apologise for the lack of clarity. We have now specified more clearly that data from the first 70 patients were used to finalise the development of the modules and the pilot testing began thereafter.**

1. I suggest that research activities (eg planned feasibility study mentioned under checklist item 11 and research data collection mentioned under item 5) are separated clearly from the intervention design in the results and discussion.

**As suggested, we have now separated the planned feasibility study and research data collection from item 5.from the results. **

1. I suggest reframing the discussion to briefly describe the final intervention and how it addresses the problem; compare with other approaches in the literature; discuss the strengths and weaknesses of the development approach, and particularly how generalisable the intervention might be or what local adaptation would be required for others to trial it; and outline plans for locally evaluating the intervention now that it is mature.

**Thank you for the suggestion. We have now reframed the discussion as suggested.**
